# Sources of carbon supporting the fast growth of developing immature moso bamboo (*Phyllostachys edulis*) culms: inference from carbon isotopes and anatomy

**DOI:** 10.1093/aobpla/plad046

**Published:** 2023-07-04

**Authors:** Shitephen Wang, Daniel Epron, Keito Kobayashi, Satoru Takanashi, Masako Dannoura

**Affiliations:** Graduate School of Agriculture, Kyoto University, Kitashirakawa Oiwake-cho, Sakyo-ku, Kyoto 606-8502, Japan; Graduate School of Agriculture, Kyoto University, Kitashirakawa Oiwake-cho, Sakyo-ku, Kyoto 606-8502, Japan; Kansai Research Centre, Forestry and Forest Products Research Institute, 68 Momoyamacho Nagaikyutaro, Fushimi-ku, Kyoto 612-0855, Japan; Kansai Research Centre, Forestry and Forest Products Research Institute, 68 Momoyamacho Nagaikyutaro, Fushimi-ku, Kyoto 612-0855, Japan; Graduate School of Agriculture, Kyoto University, Kitashirakawa Oiwake-cho, Sakyo-ku, Kyoto 606-8502, Japan

**Keywords:** Anaplerotic pathway, carbon isotope fractionation, δ^13^C of carbon compounds, Phyllostachys edulis, pulse ^13^CO_2_ labelling

## Abstract

*Phyllostachys edulis* is a spectacularly fast-growing species that completes its height growth within 2 months after the shoot emerges without producing leaves (fast-growing period, FGP). This phase was considered heterotrophic, with the carbon necessary for the growth being transferred from the mature culms via the rhizomes, although previous studies observed key enzymes and anatomical features related to C_4_-carbon fixation in developing culms. We tested whether C_4_-photosynthesis or dark-CO_2_ fixation through anaplerotic reactions significantly contributes to the FGP, resulting in differences in the natural abundance of *δ*^13^C in bulk organic matter and organic compounds. Further, pulse-^13^CO_2_-labelling was performed on developing culms, either from the surface or from the internal hollow, to ascertain whether significant CO_2_ fixation occurs in developing culms. *δ*^13^C of young shoots and developing culms were higher (−26.3 to −26.9 ‰) compared to all organs of mature bamboos (−28.4 to −30.1 ‰). Developing culms contained chlorophylls, most observed in the skin tissues. After pulse-^13^CO_2_-labelling, the polar fraction extracted from the skin tissues was slightly enriched in ^13^C, and only a weak ^13^C enrichment was observed in inner tissues. Main carbon source sustaining the FGP was not assimilated by the developing culm, while a limited anaplerotic fixation of respired CO_2_ cannot be excluded and is more likely than C_4_-photosynthetic carbon fixation.

## Teaser

*Phyllostachys edulis* , a fast-growing bamboo species, undergoes a rapid growth phase without producing leaves. We found that the carbon needed for growth is mainly transferred from mature bamboos via rhizomes. We also observed the Kranz anatomical feature in developing culms as the same as in previous studies. However, our results suggest that the main carbon source for the rapid growth phase is not assimilated by the developing culm, indicating a limited role of C4 photosynthesis. Instead, a minor contribution from anaplerotic fixation of respired CO_2_ is more likely.

## Introduction

*Phyllostachys edulis* is a large monocotyledon (Poaceae, subfamily Bambusoideae) commonly distributed in East Asia’s temperate or subtropical mountains. The plant grows incredibly fast from young shoots emerging from the ground to mature bamboos (10–15 m high) within 60 days without producing leaves, the photosynthesising organs (Yen and Lee 2011). Young bamboo shoots are shoots covered by sheaths before 25 to 40 internodes about 7.5 cm in diameter simultaneously undergoing cell division (0.06 cells cell^−1^ h^−1^) and elongation (0.49 μm μm^−1^ h^−1^). The cell division zone at the base of the internode is up to 2 cm, while the elongation zone, the upper part of the internodes, is up to 12 cm (Chen *et al.* 2022). The elongation is completed within 21–25 days, resulting in the rapid growth of the young developing culms, during which time a large amount of carbon is required (Chen *et al.* 2022), after which the senescence of the sheath occurs (Chen *et al.* 2021). The young culms accumulate three-fourths of their final biomass during the initial 40 days of this fast-growing period (FGP) (Yen 2016). The carbohydrates required to support this fast growth are thought to be transported from mature bamboos (autotrophic stage) to young leafless bamboos (heterotrophic stage) via the leptomorph rhizome systems (Song *et al.* 2016; Wang *et al.* 2020; Zhai *et al.* 2022).

*Phyllostachys edulis* is a C_3_ plant, based on plastome phylogenetic trees, like rice (subfamily Oryzoideae), its close relative (Bianconi *et al.* 2020). This is confirmed by the leaf isotope composition (*δ*^13^C) of *P. edulis* (Hanba *et al.* 2010). Roots or stems are, however, expected to be slightly enriched compared to leaves because of post-photosynthetic carbon fractionations (Badeck *et al.* 2005). Early wood in the tree rings of deciduous species is often enriched in ^13^C because the carbon used in early spring is remobilised from carbon reserve (e.g. enriched starch), while the production of late wood is more directly related to the current photosynthetic carbon assimilation (Damesin and Lelarge 2003; Helle and Schleser 2004; Gessler *et al.* 2014). Although not yet studied, there are good reasons to think that developing culms of bamboos should also be enriched compared to the leaves of mature bamboos if the photosynthetic products of mature bamboos are used to build storage compounds that are then transferred to developing immature culms.

Different organs of the same species also possibly exhibit different patterns of photosynthesis even though a plant is commonly recognised as a single photosynthetic type (e.g. C_3_ or C_4_). For instance, leaves show the C_3_ type and stems show C_4_ carbon fixation in celery and tobacco (Hibberd and Quick 2002). Although previous studies have shown that C_4_ enzymes are upregulated during the heterotrophic stage of developing culms, little is known about their contribution to the carbon gain of new-born bamboo culms during the FGP. Wang *et al.* (2012) observed that the parenchyma cells around the vascular bundles in the developing immature bamboo culms contained chloroplasts. Wang *et al.* (2021) then revealed that the genes of the important enzymes related to the C_4_ carbon fixation, such as the phosphoenolpyruvate carboxylase (PEPC), the nicotinamide adenine dinucleotide phosphate-malate dehydrogenase (NADP-MDH) and the NADP-malic enzyme (NADP-ME), were upregulated during the FGP. Furthermore, they revealed the activity of these key enzymes in developing culms during the FGP, suggesting that the 2-mm-thick skin of culms operates the NADP-ME subtype of C_4_ carbon fixation. Mature *P. edulis* is therefore a typical C_3_ plant but C_4_-like patterns may be present in developing culms. Carbon fixation may therefore not only occur in leaves, the autotrophic organ of mature bamboo, but also in developing culms during the FGP, which would result in a strong ^13^C enrichment of developing culm organic matter compared to the leaves of mature bamboos.

It is well known that heterotrophic plant organs, such as tree stems, can assimilate carbon and that the source of CO_2_ is partially from respiration (Cernusak and Marshall 2000; Wittmann *et al.* 2005; Berveiller *et al.* 2007). Forty per cent of carbon loss by respiration can be reassimilated in the current-year stems of European beech for example (Damesin 2003). The ^13^C signature of the respired CO_2_ is determined by the relative contributions of the decarboxylation related to the pyruvate dehydrogenase activity and those occurring during the Krebs cycle (isocitrate dehydrogenase and alpha-ketoglutarate dehydrogenase activities), leading to an apparent fractionation of up to 6 ‰ (Ghashghaie *et al.* 2003; Tcherkez *et al.* 2003). Carbon fixation in tree stems can occur in darkness (Höll 1974; Langenfeld-Heyser 1989), which suggests the recycling of respired CO_2_ by the anaplerotic pathway, involving the PEPC and generating ^13^C-enriched organic acids due to the fractionation in favour of ^13^C, with discrimination relative to gaseous CO_2_ of about −5.7 ‰, including therefore those of the carbonic anhydrase (CA) involved in the hydration of CO_2_ (respectively 2.2 ‰ discrimination for the PEPC and −9 ‰ for the CA, Badeck *et al.* 2005; Ghashghaie and Badeck 2014).

Our objective was to clarify which carbon sources contribute to the fast-growing stage of immature culms of *P. edulis*, from the developing culm itself or exogenously from mature bamboos. We postulated that photosynthates produced by the mature bamboos are the main carbon source for the fast growth of developing bamboo culm and that, therefore, the *δ*^13^C of developing bamboo does not strongly differ from the *δ*^13^C of all organs of mature *P. edulis*. To test this hypothesis, we collected samples of various organs at both the autotrophic and heterotrophic stages and analysed their natural ^13^C abundance in the bulk organic matter and the fractions of different organic compounds. We further postulate that developing culms can nevertheless fix a limited amount of CO_2_ during the FGP. To test this hypothesis, we operated a ^13^C pulse labelling at the surface and in the hollow of the developing culm and traced the labelled carbon in the soluble polar fraction (sugars, amino acids and organic acids) in the tissues of the developing culms.

## Materials and Methods

### Experimental site

This study was conducted in a moso bamboo stand at the Katsura Campus, Kyoto University (Kyoto Prefecture, Japan; 34°59ʹ06.2ʹʹN, 135°40ʹ48.4ʹʹE, 110 m alt.). Mean annual temperature and mean annual precipitation in 2021 were 16.9 °C and 1552 mm. The stand management included rough selective cutting and bamboo shoot harvesting without fertilisation. The mean height of the culms of mature bamboos was 15.3 m and the average diameter at breast height (DBH) was 9.9 cm.

### Sample collection of isotope analysis (natural abundance)

**Figure 1.**
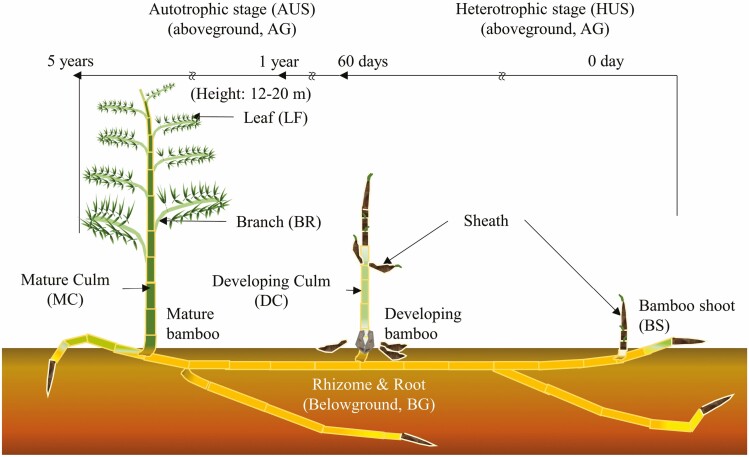
Illustrations of *P. edulis* at different growth stages. Mature bamboo culms (MC) (autotrophic stage, AUS) bear branches (BR) and leaves (LF) (aboveground, AG) and rhizomes (RH) and roots (RO) in the soil (belowground, BG). Young bamboo shoots (BS) emerge from the ground attached to mature bamboo rhizomes. Developing, immature culms grow rapidly within 3 months (heterotrophic stage, HES). Young bamboo shoots are shoots covered by sheaths before the internodes undergoing extensive elongation. Developing culms are the growing culms that undergo a period of rapid internode elongation, after which the senescence of the sheath occurs, and eventually mature into fully formed culms.

Between April and May 2021, three replicates of each organ were collected from three different individuals, including culms, branches, leaves, rhizomes and roots for mature bamboos; and bamboo shoots and developing culms for developing bamboos (Fig. 1). Samples were frozen in liquid nitrogen and first stored at −15 °C in a portable freezer in the field before being transferred to −20 °C in the laboratory.

### ^13^CO _2_ labelling experiment

The ^13^CO_2_ pulse labelling experiment was conducted on 11 May 2021. Six immature, leafless but vigorous culms of similar size were selected (mean DBH = 11.7 cm, mean height = 6 m). Three labelling chambers were installed on three developing culms around the 7th internode from the ground without sheaths (chamber size, Φ = 20 cm, height = 15 cm). The chambers were made of a transparent thin polycarbonate sheet wrapped around the culm on polypropylene half-circular plates at both the bottom and the top of the chamber, with semi-circular holes at their centre to accommodate the culm. They were affixed to the surface of the culm with neutral seal putty and a silicon sealant [see Supporting Information—**Fig. S1**]. At 13:20 h, 75 mL of 99 % ^13^CO_2_ was injected into each chamber from three directions (three times 25 mL with a needle inserted through the polycarbonate sheet). The concentration of ^13^CO_2_ in the chambers was 2.4 %, based on the volume of the chamber and the amount of ^13^CO_2_ injected. In the other three developing culms, 50 mL of 99 % ^13^CO_2_ was injected with a needle inserted into the hollow of the 7th internode without sheaths. The concentration of ^13^CO_2_ in the hollows was 3.7 %, based on the volume of the hollow and the amount of ^13^CO_2_ injected. The small pinhole was sealed by tape. Two hours after the injection of ^13^CO_2_ in either the chamber or the culm hollow, the 5th, 7th and 9th internodes of the six labelled and two unlabelled developing culms were sampled. Disks of 100–200 g were collected using a hand saw shortly after labelling to limit the proportion of labelled products potentially transported away from the assimilation site. Samples were frozen in liquid nitrogen and first stored at −15 °C in a portable freezer in the field before being transferred to −20 °C in the laboratory, as above.

### Purification of polar fractions, structural compounds, starch and proteins from different organs

In the laboratory, all samples were freeze-dried for at least 72 h using a vacuum freeze dryer (FDU-1200, EYELA, Tokyo, Japan), and ground to a fine powder in a ball mill (MM-400, Retsch, Düsseldorf, Germany).

The polar fraction, starch, proteins and structural compounds were separated and purified according to the modified methods of Deleens and Garnier-Dardart (1977), Wanek *et al.* (2001), Richter *et al.* (2009) and Desalme *et al.* (2017). These purification procedures have been developed specifically to allow the carbon isotope analysis of organic fractions. Briefly, soluble and insoluble compounds were separated based on their polar properties in a mixture of methanol–chloroform–water (12/5/3, v/v/v). After centrifugation, the supernatant was mixed with 0.5 mL of methanol–chloroform (MC, 1/1, v/v) to separate pigments and lipids in the lower phase from the polar compounds in the upper phase. The pellet was digested with pronase in Tris buffer. Proteins were solubilised while starch remains in the insoluble fraction. The insoluble fraction was washed with ethanol and starch was gelatinised by hydrochloric acid. The starch fraction, recovered in the supernatant after centrifugation, was precipitated with absolute methanol. The structural fraction was recovered in the pellet washed with water at least 3 times. All fractions were oven-dried at 65 °C and weighed. The methodology detailed step by step is available [**see Supporting Information—Method S2 and Fig. S2**].

### *δ* ^13^C determination of total organic matter, polar fractions, structural compounds, starch and proteins

For the determination of the isotope composition, 1 mg of dried samples or dried fractions was weighed in tin capsules and combusted in elemental analysers coupled to isotope ratio mass spectrometers (Thermo FLASH2000, DeltaV advantage with ConfloIV system, Thermo Fisher Scientific, Massachusetts, USA; for unlabelled samples, and Thermo NC2500, MAT252 with ConfloIII system, Thermo Fisher Scientific, Massachusetts, USA; for labelled samples). Several international standards [IAEA-CH-3 (−24.724 ‰, Coplen *et al.* 2006), USGS40 (−26.39 ‰, Qi *et al.* 2003) and USGS41a (36.55 ‰, Qi *et al.* 2016)] were used for calibrations. USGS 41a was further used as a running standard for every 11 samples. The analysis of the 11 samples would have been rerun if the running standard had been an outlier of all standards (outlier defined in Reed *et al.* 1971), which did not happen. Results (*δ*^13^C) were expressed in ‰ as the relative deviation of the isotope ratio of the sample (^13^C/^12^C, $R_{\text{sample}}$) compared to that of the international VPDB standard (Vienna PeeDee Belemnite).


$$\delta_{\;}^{13}\text{C}=\left(\frac{R_{\text{sample}}}{R_{\text{vpdb}}}-1\right)\text{ ​​}\;\text{​​ }$$ (1)

The isotope composition of the weighted fractions (*δ*^13^all) was defined as:


$$\delta_{\;}^{13}\text{all}=\frac{\sum_{\;}^{\;}\delta_{\;}^{13}\text{C}\times Cf\times Wf}{\sum_{\;}^{\;}Cf\times Wf}\;$$ (2)

with *Cf*, the carbon ratio for each fraction refers from Bowling *et al.* (2008), and *Wf*, the dry weight of fraction.

The excess amount of ^13^C in a labelled sample was calculated as:


$${\text{Excess}}_{\;}^{13}C=\left(x{\left({}_{\;}^{13}\text{C}\right)}_{\text{L}}-x{\left({}_{\;}^{13}\text{C}\right)}_{\text{UN}}\right)\times \text{C}$$ (3)

with *x*(^13^C)_L_ and *x*(^13^C)_UN_, the atom fraction of ^13^C in labelled and unlabelled samples respectively, and C, the total carbon content.

The atom fraction was calculated as:


$$x\left({}_{\;}^{13}\text{C}\right)=\frac{{}_{\text{ ​​}\;\text{​​ }}^{\text{13}}\text{C}}{{}_{\text{ ​​}\;\text{​​ }}^{\text{12}}\text{C}{\text{+}}_{\text{ ​​}\;\text{​​ }}^{\text{13}}\text{C}}=\;\frac{R_{\text{sample}}}{R_{\text{sample}}+1}$$ (4)

### Culm anatomy and starch granule observation

The 7th internode of developing culms was cut into small cubes. Cross and vertical sections 10–30 μm thick were sliced off the frozen samples (MCR802A, Komatsu Electronics, Ishikawa, Japan) with a sliding microtome (TU-213, Yamato Kohki, Saitama, Japan). Each section was loaded on a glass slide and covered carefully with a coverslip to avoid air bubbles. After initial observation, sections were rinsed with ultrapure water and stained with 0.1 N iodine solution, and briefly rinsed with ultrapure water again. Microphotograph images were taken with a digital camera (EOS KISS X3, Canon, Tokyo, Japan) attached to a light microscope (13X50-32, Olympus, Tokyo, Japan).

### Chlorophyll content measurement

Two mm wide vertical sections of the skin, middle and inner parts of the 7th internode in developing culms were cut for chlorophyll extraction. Chlorophyll was extracted from 500 mg of fresh samples using 4 mL of *N*,*N*-dimethylformamide as the extraction solvent. Extraction was conducted in darkness at 65 °C for 2 h followed by 1 h at room temperature (Berveiller and Damesin 2008). At the end of the extraction, the samples were colourless. The absorbance of the extract was measured at 470, 663.6 and 646.6 nm using a spectrophotometer (ASV-S3, As One Corporation, Osaka, Japan). The amounts of chlorophyll *a* and *b* were calculated using the extinction coefficients obtained from Porra *et al.* (1989).

### Statistical analysis

Data presented in this study are the means of three replicates from three individuals. One-way ANOVA followed by the Tukey HSD post hoc test was performed to test the between-organ differences in *δ*^13^C in bulk organic matter and in different organic fractions, and the content in these different fractions. It was also used to test the differences in chlorophyll content between the skin, middle and inner tissues of the developing culms. Univariate student’s *t*-test was used to test whether the excess ^13^C in the polar fraction was significantly higher than 0 after pulse-labelling developing culms with ^13^CO_2_. All statistical analyses were performed using R (R Core Team 2023).

## Results

### Variations in natural ^13^C abundance in bulk organic matter between organs

**Figure 2.**
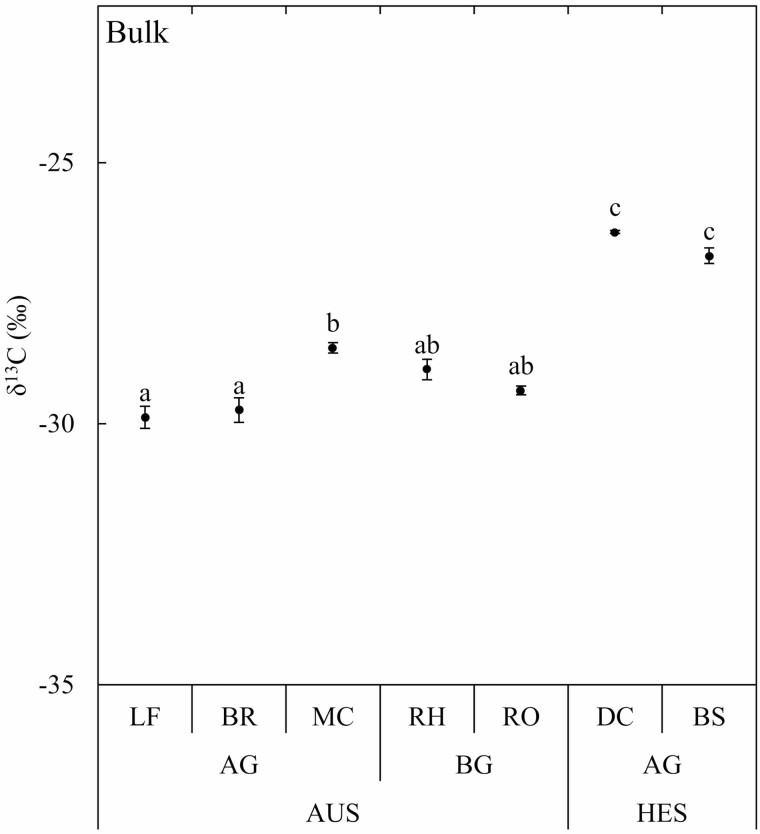
Isotope composition (*δ*^13^C) of bulk organic matter in organs of *P. edulis* at different growth stages. Average (*n* = 3) with standard errors are shown. Different letters indicate significant differences (one-way ANOVA and Tukey HSD post hoc test at *P* < 0.05) between leaves (LF), branches (BR), mature culms (MC), rhizomes (RH), roots (RO), developing culms (DC) and bamboo shoots (BS). The autotrophic stage (AUS) includes both aboveground (AG) and belowground (BG) organs of mature bamboos. The heterotrophic stage (HES) includes newly formed bamboo shoots and developing immature bamboo culms during their fast-growing period (FGP).

The isotope composition of the different autotrophic organs of mature bamboos (leaves and branches) and belowground systems (rhizomes and roots) ranged between −28.4 and −30.2 ‰ (−29.3 ‰ on average, Fig. 2). The isotope composition of culms was higher than that of mature bamboo branches and leaves (−28.5 and −29.8 ‰ on average, respectively; *P* < 0.05; Fig. 2). The bulk *δ*^13^C of bamboo shoots and developing culms (−26.8 and −26.3 ‰ on average; Fig. 2) were higher than that of all organs of mature bamboos (*P* < 0.001).

### Variations in the content of biochemical fractions between organs

**Figure 3.**
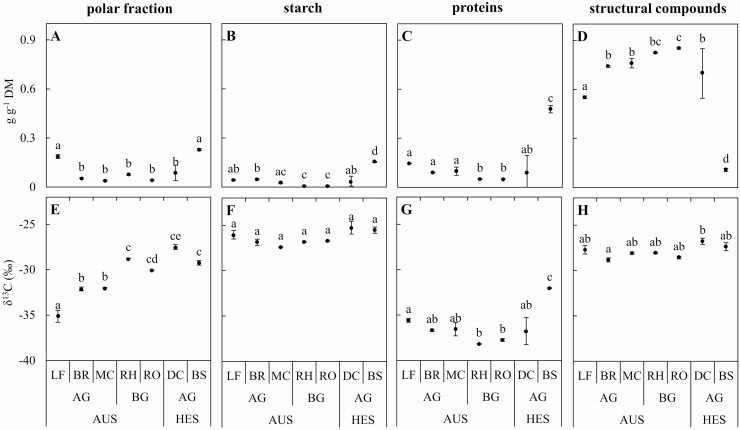
Contents and isotope carbon compositions (*δ*^13^C) of main biochemical fractions in organs at different growth stages. (A and E) Polar fraction. (B and F) Starch. (C and G) Proteins. (D and H) Structural compounds. Average (*n* = 3) with standard errors are shown. Different letters indicate significant differences among organs (one-way ANOVA and Tukey HSD post hoc test at *P* < 0.05). LF, BR, MC, RH, RO, DC and BS indicate leaves, branches, mature culms, rhizomes, roots, developing culms and bamboo shoots, respectively. The autotrophic stage (AUS) includes both aboveground (AG) and belowground (BG) organs of mature bamboos. The heterotrophic stage (HES) includes newly formed bamboo shoots and developing immature bamboo culms during their fast-growing period (FGP).

The content in the polar fraction was higher in the foliage than in other organs of mature bamboo and developing immature bamboo shoots (*P* < 0.001) but similar to that in young bamboo shoots (*P* > 0.05; Fig. 3A). The starch content was low in all organs, especially in the belowground part of the mature bamboos, except in the young bamboo shoots (Fig. 3B). More proteins were found in the aboveground organs of mature bamboos than in their belowground organs. While the protein content was not different in developing bamboo culms compared to mature bamboos (*P* > 0.1), the young bamboo shoots contained a much larger amount of proteins than all the other samples (0.48 g g^−1^ compared to 0.09 g g^−1^ on average in all other samples; *P* < 0.001; Fig. 3C). The content in structural compounds was much lower in young bamboo shoots (0.10 g g^−1^; *P* < 0.001; Fig. 3D) than in all the other organs. Low values were also observed in leaves (0.55 g g^−1^, Fig. 3D), while it averaged 0.84 g g^−1^ in the belowground organs, 0.75 g g^−1^ in branches and mature culm and 0.70 g g^−1^ in developing bamboo culms (Fig. 3D).

### Variations in natural ^13^C abundance between biochemical fractions

The polar fraction was depleted in ^13^C compared to the bulk organic matter and showed large variations among organs, from −35.1 ‰ in leaves to −27.5 ‰ in developing bamboo culms (Fig. 3E). The isotope composition of polar fractions from the rhizome (−28.8 ‰), less depleted than that of the other organs of the mature bamboos (< −30 ‰), was not different than that of the developing bamboo culms (−27.5 ‰; *p* > 0.1) and young bamboo shoots (−29.3 ‰; *P* > 0.1). In contrast to the polar fraction, the starch fraction was enriched in ^13^C compared to the bulk organic matter and much less variable (Fig. 3F). The protein fraction was strongly depleted in all organs compared to the bulk organic matter (from −35.6 to −38.2 ‰, Fig. 3G) except for the young bamboo shoots, which showed a *δ*^13^C higher value than that of all other organs (−32.0 ‰; *P* < 0.001). *δ*^13^C of the structural compounds was slightly higher in the developing bamboo culm (−26.6 ‰, Fig. 3H) than that of the organs of mature bamboos (< −27.7 ‰).

**Figure 4.**
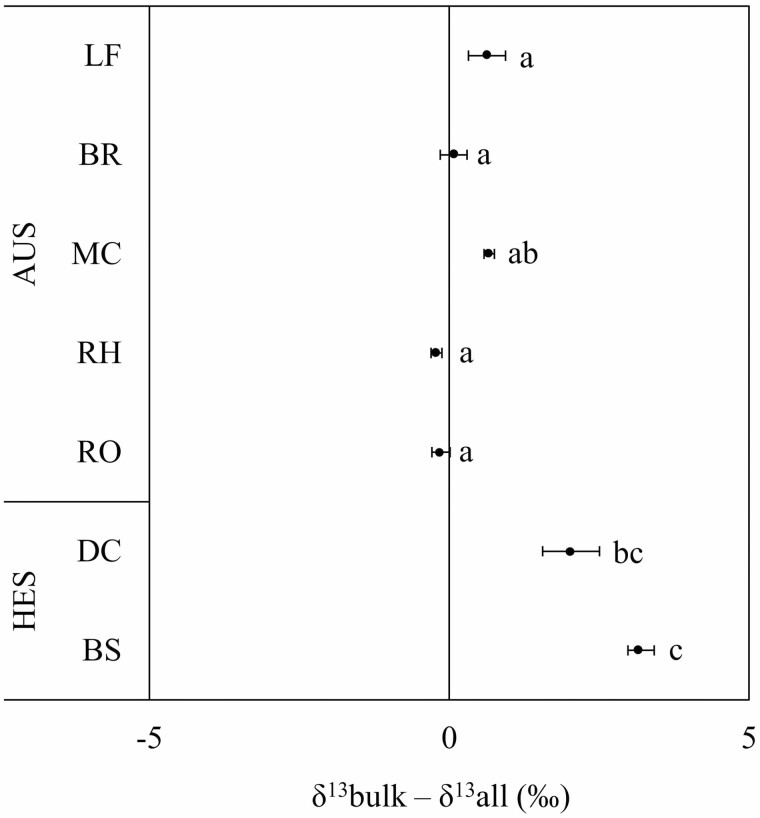
Deviation between the weighted average of the isotopic composition of the different biochemical fractions (*δ*^13^all) and the isotope composition of the bulk organic matter (*δ*^13^bulk). *δ*^13^all is the weighted average of the isotopic composition of the different biochemical fractions (polar fractions, starch, proteins and structural compounds) by their concentration and carbon content. Average (*n* = 3) with standard errors are shown. Different letters indicate significant differences among organs (one-way ANOVA and Tukey HSD post hoc test at *P* < 0.05). LF, BR, MC, RH, RO, DC and BS indicate leaves, branches, mature culms, rhizomes, roots, developing culms and bamboo shoots, respectively. The autotrophic stage (AUS) includes both aboveground (AG) and belowground (BG) organs of mature bamboos. The heterotrophic stage (HES) includes newly formed bamboo shoots and developing immature bamboo culms during their fast-growing period (FGP).

The weighted average of the isotopic composition of the different biochemical fractions (polar fractions, starch, proteins and structural compounds) by their concentration and carbon content (*δ*^13^all) was compared to the isotope composition of the bulk organic matter (*δ*^13^bulk), and the deviation can be attributed to the isotope composition of unknown compounds, especially lipids and other non-polar molecules, which have been solubilised in chloroform but not analysed isotopically due to their limited quantity. No pronounced differences between *δ*^13^bulk and *δ*^13^all were observed in mature organs (leaves, branches and culms) and belowground systems (rhizomes and roots). However, bulk organic matter in developing bamboo culms and young bamboo shoots was more enriched in ^13^C than the analysed compounds (Fig. 4).

### Chlorophyll content and anatomy of developing culms at the 7th internode

**Figure 5.**
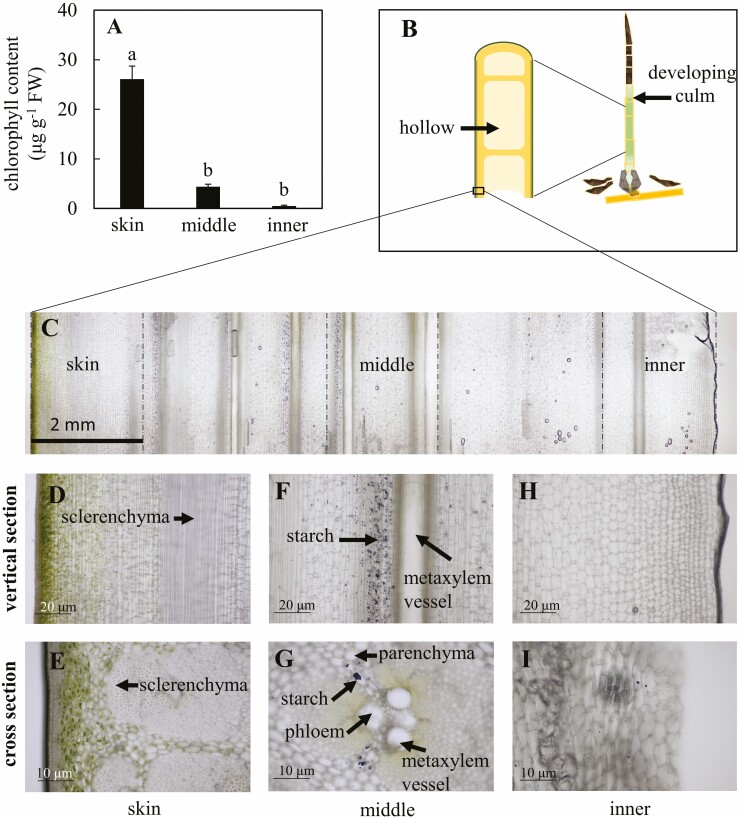
Chlorophyll content and anatomy of developing culms. (A) Average chlorophyll content with their standard errors (*n* = 6) in developing culm at different radial positions. Different letters indicate significant differences (one-way ANOVA and Tukey HSD post hoc test at *P* < 0.05). (B) Illustration of a developing culm with its internal hollow. (C) Vertical section of developing bamboo culm. (D) Vertical section of skin tissues. (E), Cross-section of the skin tissues. (F) Vertical section of tissues in the middle. (G) Cross-section of tissues in the middle. (H) Vertical section of the inner tissues. (I) Cross-section of the inner tissues.

The chlorophyll content in the skin of developing culms, which includes the layer of cortical cells, was higher than in middle and inner tissues (26.1 μg g^−1^ FW compared to 4.4 and 0.5 μg g^−1^ FW on average, respectively; *P* < 0.001; Fig. 5A). However, when expressed per unit area, the chlorophyll content in the skin was similar to that in mature leaves (respectively 5.6 and 4.2 μg cm^−2^ on average). Chlorenchyma cells were distributed around the skin of developing culms (Fig. 5C–E). In the middle part of the developing culm, chlorophyll was found preferentially near the metaxylem vessels and the phloem sieve tubes forming a bundle sheath around the vascular bundles (Fig. 5F and G). Furthermore, a lot of starch granules were observed in parenchyma cells around the vascular bundles of developing culms. In contrast, almost no starch granules were observed in the parenchyma cells of the inner part and the chlorenchyma cells of the skin part of developing culms (Fig. 5H and I).

### Distribution of labelled carbon after ^13^C pulse labelling of the 7th internode of developing culms

**Figure 6.**
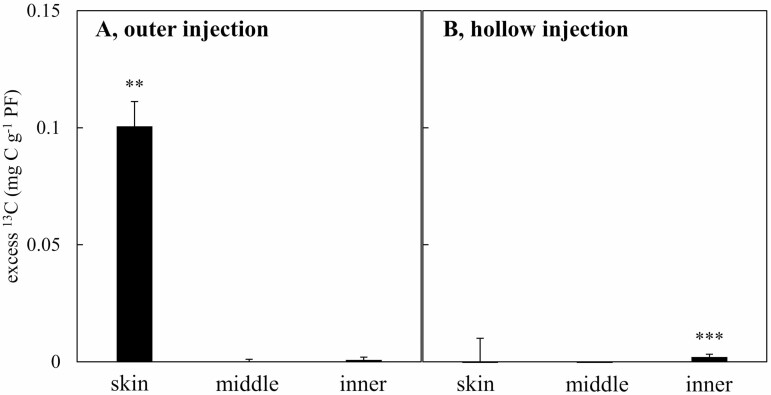
Excess ^13^C in after a pulse ^13^CO_2_ labelling at the 7th internode of developing culms during the fast-growing period. Average with their standard error (*n* = 3) of excess ^13^C in the polar fraction (PF) extracted from the skin, middle and inner tissues of the developing culms 2 h after the injection of ^13^CO_2_ in either a chamber around the culm (left, A) or the culm hollow (right, B). ‘***’ and ‘**’ indicate that the average excess ^13^C is greater than 0 (univariate student’s *t*-test at *P* < 0.001 and *P* < 0.01).

The excess ^13^C of the polar fraction (PF) extracted from the 7th internode of developing culms was significantly higher than 0 near the skin (0.1 mg ^13^C g^−1^ PF on average, Fig. 6A) while it was not significantly different from 0 deeper in the culms (0.0003 mg ^13^C g^−1^ PF in the middle and 0.001 mg ^13^C g^−1^ PF in the inner parts on average). When ^13^CO_2_ was injected in the hollow inside the 7th internode of the developing culms, little or no enrichment was recovered in any sample compared to unlabelled samples, but it was significantly different from 0 in the inner part, although the enrichment value was small (0.002 mg ^13^C g^−1^ PF, Fig. 6B). No enrichment was found in the internodes below (5th) or above (9th) the internode 7th which was labelled, confirming that the labelled carbon did not migrate up or down during the short 2-h tracing (data not shown).

## Discussion

### Only slight ^13^C enrichment of heterotrophic organs compared to leaves

The isotope composition of the different autotrophic organs of mature bamboos (Fig. 2) was similar to those of mature leaves in a previous study (−28.3 ‰, Hanba *et al.* 2010), confirming that *P. edulis* is a C_3_ plant at the autotrophic stage. The slight enrichment in ^13^C of the different heterotrophic organs of mature bamboo (culms, rhizomes and roots) compared to autotrophic organs agrees with previous findings in several studies summarised by Badeck *et al.* (2005): roots, stems or culms (grass stems) are often slightly enriched in ^13^C compared to leaves (1–2 ‰). The reasons for this phenomenon, which is often related to post-photosynthetic fractionations, are complex, and several hypotheses have been reviewed by Cernusak *et al.* (2009). Since the isotopic composition of starch was similar for all organs while differences were found for other fractions, a seasonal change in ^13^C discrimination during photosynthesis (their hypothesis 2) or preferentially nocturnal sucrose translocation (their hypothesis 3) are therefore unlikely to explain the ^13^C enrichment of immature bamboo and heterotrophic organs of mature bamboo compared to leaves. Heterogeneous carbon isotope distribution within sugars results from enzyme-dependent fractionations occurring during sugar interconversions. Because these sugars are precursors of all organic compounds in plant tissues, these fractionations impact the ^13^C distribution in plants (Rossmann *et al.* 1991; Gilbert *et al.* 2012), given that different types of biochemical substances do not have the same isotope characteristics (Bowling *et al.* 2008). The differences in the isotope composition between organs are therefore thought to be related to their differences in the biochemical composition (Cernusak *et al.* 2009, their hypothesis 1). However, although different biosynthetic pathways cause carbon-related compounds in different organs to have different ^13^C, the remaining part of the biosynthesis of a certain compound is used in the biosynthesis of other compounds, so the bulk *δ*^13^C of the organ should not be determined by internal compounds but by the balance between carbon input and output (Badeck *et al.* 2005; Gilbert *et al.* 2012). The fact that the assimilates transported from the leaves to the remaining part of the plant (input) are enriched in ^13^C due to post-photosynthetic fractionations associated with mitochondrial respiration occurring in the leaves (Damesin and Lelarge 2003; Gessler *et al.* 2007; Kodama *et al.* 2008) can therefore explain the observed slight enrichment of these heterotrophic organs (Cernusak *et al.* 2009, their hypothesis 4). Also, additional fractionations related to incomplete decarboxylation during respiration (output) can release depleted CO_2_ and contribute to the enrichment of the remaining organic matter (Damesin and Lelarge 2003; Ghashghaie and Badeck 2014). The fact that the polar fraction was similarly enriched in the belowground organs of mature bamboos and in the bamboo shoots and developing culms compared to the autotrophic organs of mature bamboos supports the hypothesis that photosynthates produced by the mature bamboos and stored in the rhizome are the main carbon source for the fast growth of developing bamboo culm (Fig. 3E). This is also consistent with the fact that the starch content was very low in the belowground part of the mature bamboos, but high in the young bamboo shoots, which may have temporarily stored starch just before the onset of the FGP (Fig. 3B). The fact that the isotopic composition of starch from young shoots was similar to that of starch from leaves of mature bamboos (Fig. 3F) suggests a possible common sugar source.

It has been shown in another bamboo (*Phyllostachys nigra*) that the chemical composition of the cell wall changed with time and that lignin accumulated at a later stage of development (Chang *et al.* 2013). It is therefore possible that the lower *δ*^13^C values in mature bamboo culms are related to a high proportion of lignin in their structural fraction compared to immature bamboos. Lignin is indeed depleted in ^13^C compared to cellulose or other structural polysaccharides (Bowling *et al.* 2008) because lignin precursors are produced by the shikimate pathway, which is fuelled by the depleted products of the pyruvate dehydrogenase and the Krebs cycle (Gessler *et al.* 2014). However, the isotope composition of the structural compounds was very similar in developing culms, bamboo shoots and organs of mature bamboos (Fig. 3H). It is therefore unlikely that the observed depletion of mature culms relative to the immature ones was related to lignin accumulation with ageing.

### Photosynthesis in new-born bamboo culms during the FGP

Huge amounts of carbohydrates should be either produced or transported from mature bamboos to meet the high demand needed to support cell elongation in the internodes of the immature bamboo culms during the period of rapid growth. Using ^13^C-enriched CO_2_ labelling, we found direct evidence that the skin of developing culm slightly uptakes the CO_2_ from the atmosphere and synthesises carbohydrates (Fig. 6A). However, because the concentration of CO_2_ in the labelling chamber was much higher than in the atmosphere (2.4 %), our results revealed a potential rather than the actual CO_2_ fixation. In addition, the estimated rate of CO_2_ fixation by the immature culms based on excess ^13^C in the polar fraction, which was about 0.1 µmol m^−2^ s^−1^, was more than 100 times lower than expected leaf photosynthesis (Zachariah *et al.* 2016) and 20 times lower than the respiration of young culms (Uchida *et al.* 2022). Our results are therefore consistent with the low chlorophyll content in the skin of developing culms compared to leaves (Fig. 5A) and the observed translocation of photosynthates from mother to daughter ramets of moso bamboo quantified using ^13^C–CO_2_ pulse labelling (Zhai *et al.* 2022).

Previous studies have also pointed out that the developing culms of *P. edulis* activated the key enzymes of C_4_ carbon fixation (such as NADP-ME, NADP-MDH, pyruvate phosphate dikinase [PPDK] and PEPC) during the FGP (Wang *et al.* 2012, 2021). A C_4_ plant-like Kranz anatomy has also been evident in the middle of the developing bamboo culm in this study (Fig. 5F and G), confirming previous observations (Wang *et al.* 2012). Another hypothesis to explain the slight enrichment of the bamboo shoots and developing culms is therefore that the PEPC is involved in a limited carbon fixation by immature culms during their initial growth, like C_3_–C_4_ intermediate species (Lundgren *et al.* 2016). PEPC refixation may contribute with malate to the Krebs cycle, thus resulting in ^13^C-enriched respired CO_2_ (Cernusak *et al.* 2009, their hypothesis 5; Gessler *et al.* 2009). However, based on direct evidence from our ^13^CO_2_ pulse labelling, the assimilation of atmospheric CO_2_ seems to be limited to the skin of the culm, as only the tissue near the skin was labelled when ^13^CO_2_ was provided around the culm, while the C_4_ plant-like Kranz anatomy was found in the middle part of the culm. In addition, the isotope composition of the starch in developing culms and bamboos shoots was not different than that in any organ of mature bamboos, which definitely excludes significant C_4_-like photosynthetic carbon fixation in immature culms (Fig. 3F).

The presence of a limited amount of chlorophyll and a large number of starch granules in the parenchyma cells around the vascular bundle in the middle part of the culm nevertheless suggests that CO_2_ assimilation can occur, the CO_2_ being potentially supplied by the xylem sap rather than diffusing from the atmosphere or the internal hollow because of limited diffusion of CO_2_ within the dense culm tissues. A high amount of water flows through the rhizome to supply the fast-growing shoots and culms with water and nutrients (Zhang *et al.* 2020). Berveiller & Damesin (2008) found that the activities of PEPC and NADP-ME were up to 13 and 30 times higher in stems than in leaves of European beech. High PEPC activity can supply malate to the NADP-ME enzyme which, in turn, can supply CO_2_ for Rubisco after decarboxylation, recycling CO_2_ derived internally from respiration, as it was postulated in stems of tobacco, celery and mikania that have characteristics of C_4_ photosynthesis in stems and exhibited a C_4_ plant-like Kranz anatomy (Hibberd and Quick 2002). However, it remains unclear whether enough light can reach the photosynthetic pigments in the bundle sheath and provide enough energy to sustain a C_4_-like carbon fixation under the deep shade conditions that prevail in the understory of a *P. edulis* because the C_4_ photosynthesis became unfavourable at low PPFD (Bellasio and Farquhar 2019). In addition, the starch in the numerous granules should have been enriched compared with CO_2_ derived from the oxidation of imported sugars if a large fraction of respired CO_2_ was reassimilated by the PEPC and used in gluconeogenesis. This is because the fractionation in favour of ^13^C by the CA involved in the hydration of CO_2_ is higher than the small fractionation against ^13^C by the PEPC (Badeck *et al.* 2005; Ghashghaie and Badeck 2014). Such enrichment was not observed in starch (Fig. 3F), suggesting that the abundant recycled CO_2_ was not used for starch synthesis but for other purposes. This again suggests that the starch was instead likely synthesised from sucrose produced in mature bamboo, transported in the phloem through the rhizomes and transiently stored in granules near elongating cells (Li *et al.* 2022). On the other hand, carbon derived from the breakdown of stored starch in mature bamboo organs should be ^13^C-enriched, as compared to primary assimilates, because starch breakdown produced ^13^C-enriched sucrose (Gessler *et al.* 2008). Therefore, a higher contribution of stored carbon to boost developing culms may lead to enriched ^13^C organic matter in immature culms.

### Anaplerotic fixation of respired CO _2_ in immature bamboo culms during the fast growth period

The inner part of the culms was slightly labelled when ^13^CO_2_ was provided in the culm hollow (Fig. 6B). This may reveal that carbon fixation is taking place in the inner parts. It has been shown that carbon fixation by the PEPC can occur in darkness in some tree stems (Höll 1974; Langenfeld-Heyser 1989) and can be stimulated in the presence of nitrate and ammonium (Müller *et al.* 1991; Vuorinen and Kaiser 1997). The CO_2_ concentration inside culms of moso bamboos, which varies during the day and over the season from 40 000 to more than 100 000 ppm (Toshihiko Maitani, unpublished results), is in a similar range as for other bamboo species (Kiyama 1905; Zachariah *et al.* 2016). Therefore, it is also possible that PEPC and NADP-ME are involved in the anaplerotic fixation of respired CO_2_ in the middle and inner culms where light cannot reach, providing the carbon skeleton (malate and oxaloacetate) necessary for amino-acid (Berveiller and Damesin 2008) and lipid syntheses (Smith *et al.* 1992), which are also needed during the FGP of bamboo. As explained above, because the fractionation in favour of ^13^C by the CA involved in the hydration of CO_2_ is higher than the small fractionation against ^13^C by the PEPC, the organic acids issued from the anaplerotic pathway are expected to be enriched. The discrepancy between *δ*^13^bulk and *δ*^13^all (weighted average of the isotopic composition of the four biochemical fractions analysed) in developing bamboo culms and young bamboo shoots (Fig. 4), which can be attributed to the isotope composition of lipids and other non-polar molecules as mentioned above, suggests that ^13^C-enriched malate, involved in the fatty acid synthesis, is partly derived from the anaplerotic fixation of CO_2_. The impact of the anaplerotic dark CO_2_ fixation on the enrichment of plant tissues also depends on how much respired CO_2_ can be reassimilated (Nalborczyk 1978). The use of ^13^C-enriched organic acids issued from the anaplerotic pathway for nitrogen assimilation may explain the slight enrichment of the abundant proteins extracted from bamboo shoots, and why this enrichment starts to vanish when the new culm is developing (Fig. 3G). While most nitrogen needed to support the fast growth is potentially remobilised from the mature bamboos and transferred via the rhizome (Shi *et al.* 2021, 2022; Zhai *et al.* 2022), additional nitrogen assimilation can occur.

## Conclusions

In spring, the bamboo shoots emerge, and the developing bamboos enter a phase of active and very rapid growth. Because the leaf of new-born bamboo had not yet developed, the main sources of carbohydrates during the FGP were supplied by mature bamboos and delivered through their belowground system to the developing culms, which is consistent with a slight ^13^C enrichment of the bulk organic matter of immature bamboos. Although previous studies observed key enzymes related to C_4_ carbon fixation, the results based on ^13^C pulse labelling showed limited CO_2_ uptake from the atmosphere and hollow during the FGP. Limited anaplerotic dark fixation of CO_2_ is more likely to occur than C_4_ photosynthetic C fixation.

## Supporting Information

The following additional information is available in the online version of this article –

**Figure S1.** Schematic illustration of ^13^CO_2_ labelling in developing culms of *Phyllostachys edulis*.

**Methods S2**. Procedure of purification for the polar fraction, structural compounds, starch and proteins from different organs in plants adjusted for *P. edulis*.

**Figure S2.** Flow chart of the laboratory analytical procedure for carbon compound purification in plant samples.

plad046_suppl_Supplementary_Material

## Data Availability

The data underlying this article are available in the Dryad repository at https://doi.org/10.5061/dryad.brv15dvf9, and the newest version of data will be updated in the Open Science Framework (OSF) repository, at https://doi.org/10.17605/OSF.IO/AVPKN

## Notes

**Plants, Ecosystems and Climate. Chief Editor:** Mary Heskel
