# Supplementary material for: Sources of carbon supporting the fast growth of developing immature moso bamboo (Phyllostachys edulis) culms: inference from carbon isotopes and anatomy
Source: AoB Plants. 2023 Jul 4;15(4):plad046. doi: 10.1093/aobpla/plad046 (PMC10368343; doi:10.1093/aobpla/plad046)

Figure S1 Schematic illustration of $^{13}CO_2$ labelling in developing culms of *Phyllostachys edulis*

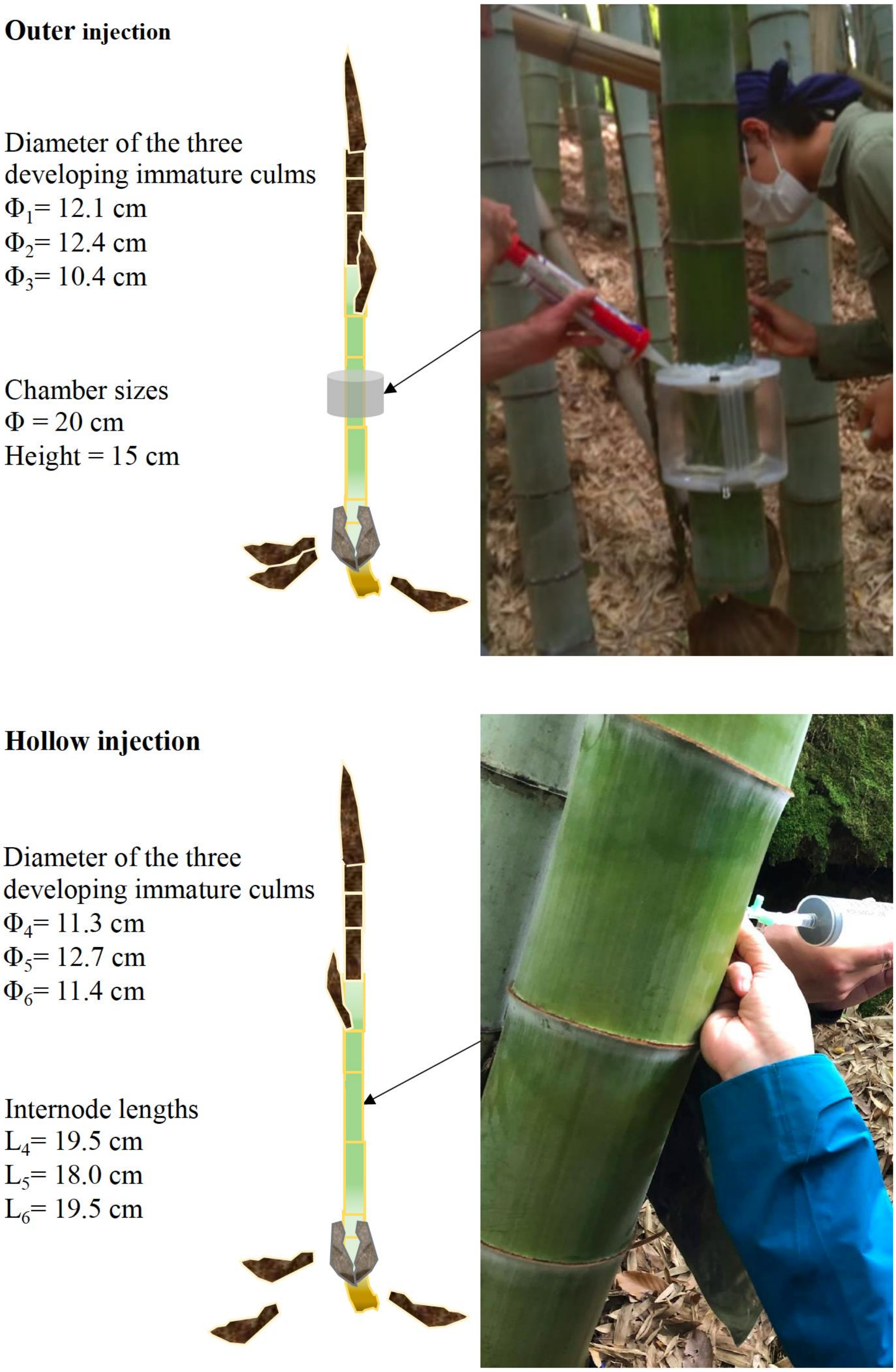

Methods S2 Procedure of purification for the polar fraction, structural compounds, starch and proteins from different organs in plants adjusted for *P. edulis*.

This procedure was adapted from Deleens & Garnier-Dardart (1977), Wanek et al. (2001), Richter et al. (2009) and Desalme et al. (2017).

List of tools and instruments:

- A centrifuge (e.g., Sorvall X Pro Series, Thermo Fisher Scientific, Massachusetts, USA).
- A microtube rotor (e.g., Fiberlite F21-48x2 Rotor, 48 x 2mL, 15,200 rpm, 25,055 x g, Φ=96.825 mm, Thermo Fisher Scientific, Massachusetts, USA).
- A rotor with adjustors of Nalgene Oak Ridge Tubes (e.g., Fiberlite F15-8x50cy Rotor, 8 x 50 mL, 14,500 rpm, 24,446 x g, Φ=103.815 mm, Thermo Fisher Scientific, Massachusetts, USA)
- A vacuum freeze dryer (e.g., FDU-1200 EYELA, Tokyo, Japan)
- A block bath (e.g., MyBL-100CS, ASONE, Osaka, Japan)
- An ultrapure water system (e.g., ADVANTEC CPW-101, TOYO ENGINEERING WORKS, Kanagawa, Japan)
- A pH meter (e.g., LAQUA D-200-1, HORIBA, Kyoto, Japan)
- A drying oven (e.g., Forced convection drying oven DO-600FA, ASONE, Osaka, Japan)
- A fridge (e.g., GALILEI URN-080RM6, Fukushima Industries, Osaka, Japan)
- A freezer (e.g., Bio Medical Freezer MDF-U339, Panasonic, Osaka, Japan)
- Oak Ridge tubes (e.g., Nalgene® Oak Ridge High-Speed PPCO Centrifuge Tubes, 28 mL, Thermo Fisher Scientific, Massachusetts, USA)
- A pipette (e.g., Gilson Pipette 200 μL-1,000 μL, Gilson, Villiers-le-Bel, France)
- A vortex (e.g., TUBE MIXER TRIO TM-1N, ASONE, Osaka, Japan)
- Microbalance (e.g., HR-200, A&D, Tokyo, Japan)
- Microtubes (e.g., Cliklok low surface tension microcentrifuge tubes 2 mL T330-72LST, Simport Scientific Inc., Quebec, Canada)
- Tips (e.g., Standard Tip 1,000 μL 110-706C, WATSON Bio Lab, Kobe, Japan)

List of chemicals:

- $CH_3OH_{(aq)}$ ≧99.0% (e.g., Methanol 67-56-1, Nacalai, Kyoto, Japan)
- $CHCl_{3\,(l)}$ ≧98.0% (e.g., Chloroform 67-66-3, Nacalai, Kyoto, Japan)
- $NH_2C(CH_2OH)_3$ (s) ≥99.8% (e.g., Tris (hydroxymethyl) aminomethane 77-86-1, Sigma-Aldrich, Massachusetts, USA)
- $HCl_{(aq)}$ 35~37% (e.g., Hydrochloric acid 7647-01-0, Nacalai, Kyoto, Japan)
- pronase >4000 U mg^{-1} (e.g., pronase E 9036-06-0, Santa Cruz, Texas, USA)
- $C_2H_5OH_{(aq)}$ ≧99.5% (e.g., Ethanol 64-17-5, Nacalai, Kyoto, Japan)

Step 0. Initial preparation

0.1. Pre-dry all 2 mL centrifuge tubes at 65°C for 72 h.

0.2. Add names to the 2 mL centrifuge tubes: IA & IB for structural compounds, IIA & IIB for lipids and pigments, IIIA & IIIB for the polar fraction (PF), IVA & IVB for proteins and parts of insoluble pigments, and V for starch. Record the weight of all tubes. [We recommend preparing at least two control checks from I to V in each trial]

0.3. Add 50 mg of the sample powder to tubes IA & IB, respectively, then record the weight again. Put tubes IA & IB in an oven at 65°C for 24 h, then record weight again.

[We recommend preparing four tubes for mature culms, rhizome, and roots of *P. edulis* (200 mg totally) in summer due to their very low starch content]

[We recommend using only 30 mg for bamboo shoots due to their high starch, PF and proteins content]

0.4. Mix 96 mL of methanol ($CH_3OH_{(aq)}$), 40 mL of chloroform ($CHCl_{3\,(l)}$) and 24 mL of deionised (DI) water in a 200 mL beaker, transfer to a 500 mL glass bottle, and name it MCW solution for step 1.

0.5. Mix 50 mL of methanol ($CH_3OH_{(aq)}$) and 50 mL of chloroform ($CHCl_{3\,(l)}$) in a 200 mL beaker, transfer to a 500 mL glass bottle, and name it MC solution for step 1.

0.6. Dissolve 1.21 g of tris(hydroxymethyl)aminomethane in 150 mL of DI water in a 300 mL beaker, adjust pH at 7.4 with 1M $HCl_{(aq)}$, mix well, transfer to a 200 mL volumetric flask, top up with DI water to 200 mL, and name it tris-buffer (0.05M, pH7.4).

0.7. Transfer 65 mL of the tris-buffer to a 200 mL beaker and add 26 mg of pronase (Actinase E from Streptomyces griseus), mix well and name it tris-pronase buffer for step 3.

0.9. Add 100 mL of DI water to a 300 mL beaker and 100 mL of 37% (12N) of hydrochloric acid ($HCl_{(aq)}$) and transfer the mixture to a 300 mL glass bottle and name it HCl (6N) for step 5.

Step 1. Soluble and insoluble carbon separation

1.1. Add 400 µl of the MCW mixture to tubes IA & IB on the dry powder and mix using a vortex for 15 s.

1.2. Wait for 30 min at an ambient temperature around 20-25°C and centrifuge at 2,000 g for 10 min.

[If the ambient temperature is lower than this range, the solubility of soluble sugar will decrease, and the extraction rate may decrease as well]

1.3. Transfer the supernatant from tubes IA & IB to tubes IIA & IIB, respectively.

1.4. Repeat step 1.1-1.3 twice with 200 µL of MCW. Tubes IIA & IIB contain approximately 700-800 µL of colourful supernatant for ready for step 2.

1.5. Dry the pellets in tubes IA & IB in a fume hood before starch purification in step 3.

Step 2. Separation of the polar and nonpolar fractions

2.1. Add 1 mL of the MC mixture to tubes IIA & IIB, close them, and gently flip 5 times.

2.2. Open the tubes IIA & IIB, add 400 µL of ID water, gently flip 15 times until temporary emulsification happened, and keep at room temperature for 20 min.

2.3. Centrifuge the tubes IIA & IIB at 2,000 g for 10 min and transfer the upper layer to tubes IIIA & IIIB (containing PF). Place the open tubes IIA & IIB (non-polar fraction) in a fume hood for 24 h, move them to the oven at 65°C for 24 h until they dry, and weigh them.

2.4. Place the open tubes IIIA & IIIB (containing PF) in a fume hood for 24 h, move them to the oven at 65°C for 72 h until they dry, and weigh them. Keep tubes IIIA & IIIB in a fridge at -20 °C until further $\delta^{13}C$ ratio measurements.

Step 3. Removing soluble and insoluble proteins

3.1. Add 2 mL of tris-pronase buffer to the tubes IA & IB (obtained from step 1) and vortex until the solid part is dispersed.

3.2. Incubate the tubes IA & IB for 24 h at 30°C in a block bath.

3.3. Centrifuge the tubes IA & IB at 1,200 g for 10 min.

3.4. Transfer supernatant in tubes IVA & IVB (containing polypeptides and amino acids from digested proteins). Place the open tubes IVA & IVB in a fume hood for 24 h, move them to the oven at 65°C at least for 72 h until they dry, and weigh them.

3.5. Keep tubes in the fridge at -20 °C until further $\delta^{13}C$ ratio measurements.

Step 4. Washing residuals

4.1. Place tubes IA & IB on the rack with cold packs.

4.2. Add 0.5 mL 80% ethanol in tubes IA & IB, vortex them for 15 s, and place them in a block bath at 70°C for 10 min.

4.3. Centrifuge at 12,000 g at 4°C for 10 min and discard the supernatant.

4.4. Repeat step 4.1 to 4.3 at least 2 times (or more until the supernatant is as transparent as possible).

Step 5. Starch purification

5.1. Add 1 mL of $HCl_{(aq)}$ 6N to tubes IA & IB to solubilise starch. Vortex for 15 s.

5.2. Store the tubes for 1 h at room temperature (around 20-25°C), centrifuge at 12,000 g at 4°C for 30 min, and transfer the supernatant to 28 mL Oak Ridge polypropylene copolymer (PPCO) centrifuge tubes.

5.3. Repeat step 5.1 and 5.2 once.

Step 6. Precipitation of starch

6.1. Add 16 mL of 100% methanol to the PPCO centrifuge tubes (i.e. 4 times volume more than the total volume of IA & IB supernatants).

6.2. Store the tubes for one night (over 8 h) at 4°C.

6.3. The next morning, centrifuge at 15,000 g at 4°C for 30 min. Gently remove the supernatant with a plastic pipette from top to bottom but keep a small residual supernatant (around 1.5-2 mL).

6.4. Vortex the pellet and residual supernatant well around 15 s, and transfer the whole part using a plastic pipette in tube V (2 mL).

6.5. Centrifuge the tube V at 15,000 g at 4°C for 10 min.

6.6. Use the supernatant to rinse the PPCO centrifuge tube and transfer it back to tube V again.

6.7. Centrifuge again the tube V at 15,000 g at 4°C for 5 min.

6.8. Remove the supernatant and wash the starch with ID water 1 mL 3 times, place the open tube V (starch) in a fume hood for 24 h, move them to the oven at 30°C for 24 h, and at 65°C for 24 h until they dry, and weigh them.

Step 7. Structural compounds

7.1. Wash the pellets obtained from step 5 in tubes IA & IB by ID water 3 times, centrifuge at 15,000 g for 5 min.

7.2. Place in the oven at 65°C for 72 h, and weigh them.

References:

Deleens E, Garnier-Dardart J. 1977. Carbon isotope composition of biochemical fractions isolated from leaves of Bryophyllum daigremontianum berger, a plant with crassulacean acid metabolism: Some physiological aspects related to CO_2 dark fixation. *Planta* **135(3)**: 241–248. doi: 10.1007/BF00384896

Desalme D, Priault P, Gérant D, Dannoura M, Maillard P, Plain, C, Epron D. 2017. Seasonal variations drive short-term dynamics and partitioning of recently assimilated carbon in the foliage of adult beech and pine. *New Phytologist* **213(1)**: 140–153. doi: 10.1111/nph.14124

Richter A, Wanek W, Werner RA, Ghashghaie J, Jäggi M, Gessler A, Brugnoli E, Hettmann E, Göttlicher SG, Salmon Y et al. 2009. Preparation of starch and soluble sugars of plant material for the analysis of carbon isotope composition: a comparison of methods. *Rapid Communications in Mass Spectrometry* **23**: 2476–2488. doi: 10.1002/rcm.4088

Wanek W, Heintel S, Richter A. 2001. Preparation of starch and other carbon fractions from higher plant leaves for stable carbon isotope analysis. *Rapid Communications in Mass Spectrometry* **15(14)**: 1136–1140. doi: 10.1002/rcm.353

Figure S2 Flow chart of the laboratory analytical procedure for carbon compound purification in plant samples

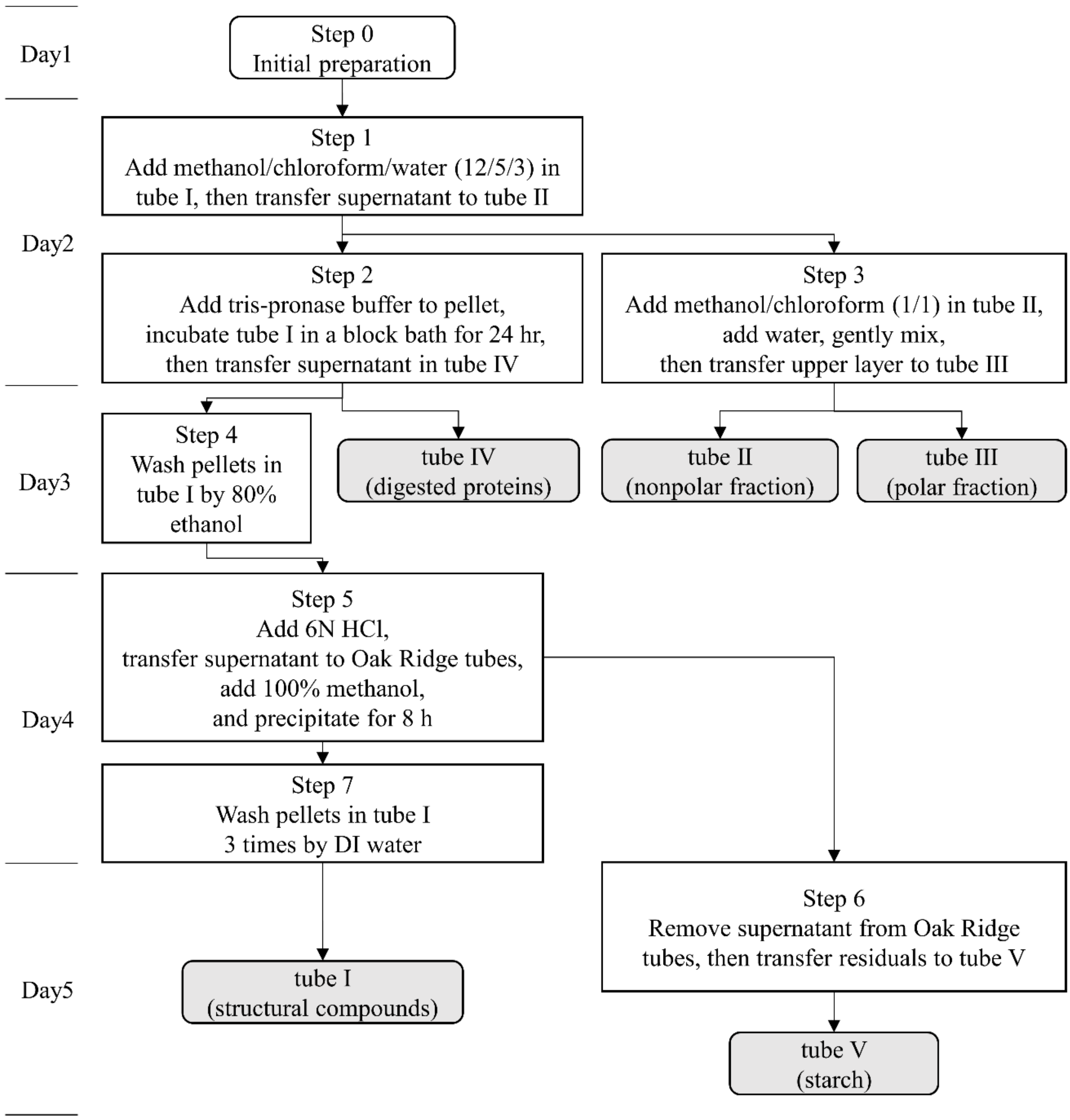

Supplement: plad046_suppl_Supplementary_Material [file plad046_suppl_supplementary_material.pdf]
